# 

**Published:** 1856-09

**Authors:** 


*To Subscribers.*—We are in receipt of numerous complaints of failure on our part to acknowledge moneys received. All those who *do not* receive a printed circular with this number, asking them to pay up, may take it for granted that if they are owing us, it is some sum less than a dollar, and probably nothing. Those who *do* receive the circular are vehemently requested to recollect that we do n't dun for the fun of it, but because we cannot afford to have some two thousand dollars constantly in arrears.

*Physician's Visiting List.*—Messrs. Lindsay & Blakiston are promptly in the field with their excellent Visiting List. On a previous page we have noticed our attempt to improve upon it. A glance at the familiar form of their "List" warns us that the task is no easy one, and that he who improves it will still be compelled to retain many of its essential features.

Well! well! We can only hope the pages of the Visiting List for 1857, may not present so many vacancies as that of this intensely healthy year.

